# Primary Dural Spinal Lymphoma Presentation of a Rare Spinal Tumor Case

**DOI:** 10.1155/2015/639253

**Published:** 2015-06-23

**Authors:** Dilber Ayçiçek Çeçen, Necati Tatarlı, Hikmet Turan Süslü, Selçuk Özdoğan, Nagehan Özdemir Barışık

**Affiliations:** ^1^Department of Neurosurgery, Dr. Lütfi Kırdar Kartal Education and Research Hospital, 34852 Istanbul, Turkey; ^2^Department of Pathology, Dr. Lütfi Kırdar Kartal Education and Research Hospital, 34852 Istanbul, Turkey

## Abstract

*Background.* Primary spinal dural lymphomas (PSDL) are tumors with characteristic histopathology of a lymphoma, which are completely in the spinal epidural space without any other systemic involvement. Extranodal primary lymphoma involving nervous system prefers thalamus/basal ganglia, periventricular region, cerebellum, eyes, meninges/dura, and cranial nerves or spinal cord. Rare spinal localization with acute spinal cord compression is worth attention. *Case Presentation.* A 48-year-old male presented with a several-month-long history of upper back pain. Lately, he had numbness and weakness at both lower extremities and was unable to walk for one week. A spinal MRI showed a thoracic lesion with cord compression at T2–T4 levels. The patient underwent surgical decompression, with his final histopathology showing diffuse large B-cell lymphoma. Systemic work-up was negative for nodal disease. Following surgery, he received radiotherapy combined with chemotherapy. He experienced a good outcome after four years. *Conclusion.* The upper thoracic cord is a rare location for primary spinal lesions/metastases, both of which prefer the lower thoracic and upper lumbar regions. In cases of progressive paraparesis, there should be immediate surgical intervention in the case of denovo disease, followed by combined radiotherapy and chemotherapy procedures.

## 1. Introduction

Spinal dural involvement in lymphoma is an entity diagnosed with secondary progression of systemic or other organs' disease which is metastatic primary lymphoma dissemination [1, 2]. Solely spinal dural lymphoma invading epidural/extradural region (which is also hardly distinguishable from dura) implying diffuse large B-cell lymphoma DLBCL (non-Hodgkin B-cell lineage) is less common [1–4] than cerebral dural lymphoma where marginal zone lymphoma is predominant [4]. Lymphoid tissue is absent in the dura, the pathogenesis of PDL is still unclear, and many hypotheses have been formulated including the role of chronic inflammatory process, chronic infection, autoimmune disease, and the meningoepithelial component [5–8]. Few case reports with other histological subtypes have been described including non-Hodgkin, Hodgkin, and marginal zone lymphoma (follicular) types [3–6, 9]. Our case reported herein is diagnosed as DLBCL of the dura matter based on the histologic and immunohistochemistry features of the malignant cells found only in the dura matter as well as the absence of systemic and CNS parenchymal involvement.

## 2. Case Presentation

A 48-year-old male presented with a six-month history of upper back pain in the dorsal T1–T5 vertebral spine region. He received some conservative medical therapy, but his complaints worsened during the previous four weeks, and he was unable to walk for the previous week. He was admitted to the emergency department with motor weakness (motor strength at lower extremities 2/5) and urgent micturition. He showed no signs of a systemic disease on either his physical examination or his laboratory findings.

**Figure 1:**
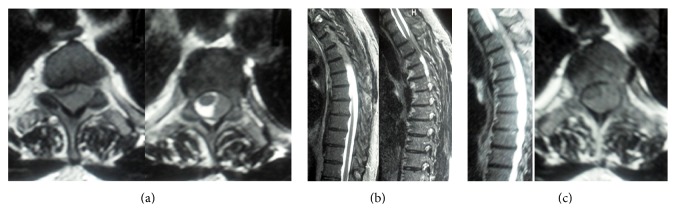
(a) Axial T2-weighted MRI shows thoracic epidural lesion with cord compression at the T2–T4 levels, extending to the paraspinal muscles. (b) Sagittal T2-weighted MRI shows dural mass. (c) Images showing T2-weighted sagittal and axial MR scans.

A spinal MRI showed a thoracic epidural lesion with cord compression at the T2–T4 levels, extending to the paraspinal muscles (Figures 1(a), 1(b), and 1(c)).

The patient underwent emergency surgical decompression with a posterior laminectomy, which showed healthy bones. A gentle excision of the pink-grey solid extradural mass could hardly be isolated from the dura.

**Figure 2:**
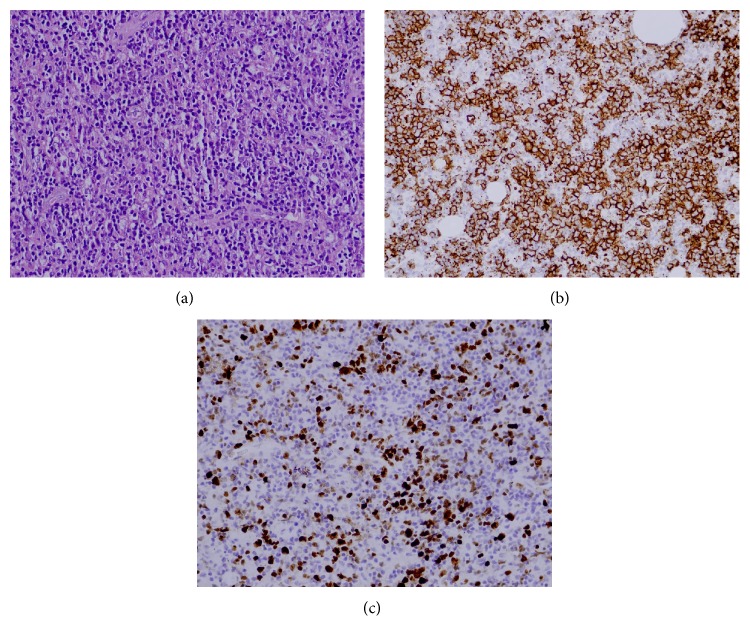
(a) Large atypical cells between small lymphoid cells (HE ×400). (b) CD 20 positivity (×400). (c) Ki 67 proliferation index was 60% (×400).

*Histopathological Studies.* Pleomorphic large vesicular cells scattered randomly or grouped which have immunoblastic morphology with significant nucleolus between small lymphocytic and histiocytic cells were demonstrated (Figure 2(a)). Immunohistochemical examination revealed CD 20 positivity in these large cells (Figure 2(b)). Ki 67 proliferation index was 60% (Figure 2(c)). Histopathological studies resulted in a diagnosis of diffuse large B-cell lymphoma.

A systemic work-up and PET-CT scans did not show evidence of a nodal disease. The patient recovered fully after the operation, and following surgery, he received a course of radiotherapy combined with three doses of chemotherapy. He has shown no progress at a follow-up during the fourth year after the operation.

## 3. Discussion

Spinal primary dural lymphoma (PDL) is uncommon, with a total of 38 well-documented case reports [4]. The most common masses seen at the upper thoracic region include primary bone tumors, metastasis, multiple myeloma, and nonspecific pyogenic infections (*Staphylococcus aureus* infections (60%),* Enterobacter*,* Salmonella*,* Klebsiella*,* Pseudomonas*, and* Serratia*). Specific infections can also affect this area, such as brucellosis, tuberculosis, Pott's disease, nocardiosis, actinomycosis, syphilis, fungal infections (coccidioidomycosis, blastomycosis, cryptococcosis, candidiasis, and aspergillosis), and parasitic infections (cysticercosis,* Echinococcus granulosus*, and schistosomiasis). Diabetes mellitus, chronic steroid use, cancer, chemotherapy, chronic renal failure, and alcohol consumption can cause spinal infections. Our study demonstrated no associated substance use or systemic diseases that would affect immune competence [5].

In 119 patients with primary bone lymphoma, surgical intervention was needed for pathologic fractures, avascular necrosis, spinal cord compression (2 cases), or lesions on the weight-bearing bones, which compromise stability and/or joint motion [6]. Mneimneh et al. [4] reviewed 38 cases involving the dural spinal cord in which the thoracic region was prominent and described primary dural lymphoma in 2 cases.

In differential diagnosis (before meticulous imaging work-up showing the compartment of the spinal thoracic mass) of a thoracic mass, considering meningeal dissemination of PCNSL or an extranodal non-Hodgkin's lymphoma which accounts for 5% to 7% of primary brain tumors and 1% to 2% of all cases of non-Hodgkin's lymphoma, respectively, should be kept in mind [1, 7]. The spinal cord is the rarest site of involvement in patients with PCNSL, although meningeal dissemination to the spinal cord from an intracranial focus may sometimes occur at an advanced stage of systemic lymphoma.

The entity of primary spinal dural lymphoma is totally different from spinal cord lymphoma both in its characteristic and prognostic features which are more favorable and also different from cranial dural lymphoma by its histopathologic features which show more insidious course [4, 8, 10–12].

We observed no systemic characteristics or organ involvement other than the thoracic spine during presentation or follow-ups (12 months and 24 months, resp.). Several treatment modalities have been employed for these patients: combined modality therapy with whole brain radiation therapy (WBRT), systemic chemotherapy alone, and intrathecal (IT) chemotherapy [13]. The patient received a course of radiotherapy and three treatments of MTX chemotherapy and experienced a good outcome after four years.

## 4. Conclusion

Primary thoracic dural lymphoma should be considered to be a differential diagnosis of a thoracic epidural mass compressing the cord epidurally, even in younger patients with no signs of any other systemic illnesses.
